# Photoplethysmogram (PPG)-Based Biometric Identification Using 2D Signal Transformation and Multi-Scale Feature Fusion

**DOI:** 10.3390/s25154849

**Published:** 2025-08-07

**Authors:** Yuanyuan Xu, Zhi Wang, Xiaochang Liu

**Affiliations:** 1 School of Electronic Information and Electrical Engineering, Yangtze University, Jingzhou 434023, China; 2023710650@yangtzeu.edu.cn; 2 School of Information Engineering, Hubei University of Economics, Wuhan 430205, China; xcliu@hbue.edu.cn; 3 Hubei Key Laboratory of Digital Finance Innovation, Hubei University of Economics, Wuhan 430000, China

**Keywords:** biometric recognition, photoplethysmography, residual neural network, 2D signal transformation, feature fusion

## Abstract

Using Photoplethysmogram (PPG) signals for identity recognition has been proven effective in biometric authentication. However, in real-world applications, PPG signals are prone to interference from noise, physical activity, diseases, and other factors, making it challenging to ensure accurate user recognition and verification in complex environments. To address these issues, this paper proposes an improved MSF-SE ResNet50 (Multi-Scale Feature Squeeze-and-Excitation ResNet50) model based on 2D PPG signals. Unlike most existing methods that directly process one-dimensional PPG signals, this paper adopts a novel approach based on two-dimensional PPG signal processing. By applying Continuous Wavelet Transform (CWT), the preprocessed one-dimensional PPG signal is transformed into a two-dimensional time-frequency map, which not only preserves the time-frequency characteristics of the signal but also provides richer spatial information. During the feature extraction process, the SENet module is first introduced to enhance the ability to extract distinctive features. Next, a novel Lightweight Multi-Scale Feature Fusion (LMSFF) module is proposed, which addresses the limitation of single-scale feature extraction in existing methods by employing parallel multi-scale convolutional operations. Finally, cross-stage feature fusion is implemented, overcoming the limitations of traditional feature fusion methods. These techniques work synergistically to improve the model’s performance. On the BIDMC dataset, the MSF-SE ResNet50 model achieved accuracy, precision, recall, and F1 scores of 98.41%, 98.19%, 98.27%, and 98.23%, respectively. Compared to existing state-of-the-art methods, the proposed model demonstrates significant improvements across all evaluation metrics, highlighting its significance in terms of network architecture and performance.

## 1. Introduction

In the modern information society, personal information is widely collected and used, leading to increasingly serious issues regarding personal privacy and security. As an important means of protecting personal information security, identity recognition technology has faced more stringent demands in terms of convenience, security, and practicality. However, traditional identification methods, such as passwords and keys, have shortcomings such as being easily forgotten or vulnerable to theft. Against this background, biometric recognition technology has emerged [1,2].

Biometric recognition technology identifies individuals based on their physiological and behavioral characteristics. Compared to traditional recognition technologies, it eliminates the need for users to remember secret information or carry physical objects, thereby offering greater security and convenience [3]. However, widely adopted biometric technologies such as facial recognition [4], fingerprint recognition [5], and iris recognition [6], while effective, still have certain limitations. For instance, facial recognition may fail in low-light or complex environments and is vulnerable to spoofing using photos or videos. Fingerprint recognition can be compromised by forged fingerprints, especially through techniques like 3D printing to replicate fingerprint patterns. Although iris recognition boasts high accuracy, its sensitivity to lighting conditions and stringent environmental requirements impose practical limitations in real-world applications. Therefore, in recent years, biometric research has gradually shifted from traditional recognition methods (such as fingerprint or iris recognition) to using physiological signals (such as electrocardiograms (ECG) [7] and electroencephalograms (EEG) [8]) for identity verification. These physiological signals inherently provide unique biometric characteristics that are difficult to forge. However, these methods still face two main issues: first, the recording process is relatively complex, and second, the required equipment is costly. Although these issues have been somewhat improved with advancements in monitoring device technology, they have not been completely resolved.

Photoplethysmogram (PPG) signals, as an emerging biometric trait, have gained significant attention due to their unique advantages [9]. PPG signals offer four key benefits: uniqueness, stability, ease of acquisition, and high security. First, each individual’s PPG signal has unique waveform characteristics, which stem from their physiological structure and cardiac function. As a result, PPG signals exhibit a high level of individual distinguishability [10]. Secondly, PPG signals maintain a high degree of stability throughout a person’s life. Although heart rate may be affected by emotions and physical activity, the waveform characteristics remain relatively constant. Additionally, PPG signals can be collected using Photoplethysmogram sensors on the finger or wrist [11]. The collection equipment is cost-effective, easy to use, and user-friendly. Finally, as a dynamic physiological characteristic generated within the body, PPG signals are extremely difficult to forge, significantly enhancing security. These unique advantages make PPG signals highly promising for applications in identity recognition.

In recent years, PPG-based identity recognition has gradually become a research hotspot. Early studies focused primarily on feature extraction and traditional machine learning methods. In 2003, Y. Y. Gu’s team [12] was the first to apply PPG signals to identity recognition. They collected raw fingertip PPG signals from 17 relaxed subjects, established a corresponding database, smoothed the signals, and extracted four features from each. Using Euclidean distance for classification, they achieved a 94% recognition success rate among the 17 subjects, preliminarily validating the feasibility of PPG-based identity recognition. With the increase in the number of individuals and sample sizes, researchers began to focus on feature extraction methods based on machine learning. They leveraged prior knowledge to uncover hidden patterns in the pulse using various algorithms and achieved intelligent identity recognition through different classifiers. Kavsaogl et al. [13] extracted 20 new features from the 10 s continuous signals and their second-order derivatives of five healthy subjects. Using the KNN classifier and ten-fold cross-validation, the identification accuracy reached 95%. Reşit Kavsaoğlu et al. [14] filtered the signals using an FIR filter and extracted 40 time-domain features from the first and second derivatives. The features were ranked using Euclidean distance and classified using KNN, achieving a recognition rate of 94.44% in 30 healthy individuals. Nur Azua Liyana Jaafar et al. [15] conducted experiments on 10 individuals from the MIMIC dataset, extracting discriminative features from the second-order difference waveform and PPG signals. Using Bayesian and KNN classifiers, they achieved an optimal correct recognition rate of 97.5%. In 2016, Namini et al. [16] denoised signals with a Butterworth filter, extracted 97 features from the PPG signal, selected 30 optimal ones, and evaluated the classification performance of four classifiers, achieving an Equal Error Rate (EER) of approximately 2.17%. However, these methods typically require manually designed feature extractors, which rely heavily on empirical knowledge and involve a certain degree of subjectivity. Furthermore, artificially constructed features may fail to accurately capture the intrinsic characteristics of PPG signals, thereby degrading recognition performance.

With the rise in deep neural networks, research on PPG-based identity recognition has gradually shifted toward the field of deep learning [17,18]. Deep learning methods do not rely on manually designed features and can automatically learn complex patterns in signals, thereby improving recognition performance. Luque J et al. [19] proposed an end-to-end neural network architecture for user identity verification tasks, using convolutional neural networks (CNNs) to analyze PulseID data (43 subjects) and Troika data (20 subjects). In 1 s trials, the method achieved AUC values of 78.2% and 83.2%, respectively. However, the relatively simple CNN architecture adopted by Luque J et al., while reducing computational complexity to some extent, may struggle to fully capture the intricate and dynamic features of PPG signals, potentially missing critical information. Biswas et al. preprocessed PPG signals using a 1–18 Hz Butterworth filter and employed a two-layer 1D-CNN combined with a two-layer LSTM network for heart rate estimation and biometric recognition. On the SPC dataset, their model achieved a five-fold cross-validation accuracy of 96% [20], but the performance was relatively low, with an F1 score of 72% and precision of 67%. Eugene Lee et al. [9] adapted the MobileNet network for one-dimensional data and achieved good classification results on a 10-subject Troika dataset and a private dataset. However, their approach merely modified MobileNet to fit one-dimensional data, which, while capable of processing 1D PPG signals, has limitations in capturing the complex features of PPG signals. Hwang et al. [21] developed robust time-stability features through signal analysis and deep learning models to enhance the robustness and performance of PPG-based verification systems. Their proposed model combined CNNs and LSTM networks for PPG signal analysis, achieving an average accuracy of 87.1%. However, the study primarily focused on time-stability features, which improved the system’s adaptability to temporal variations but overlooked other important dimensions of PPG signal features, resulting in incomplete utilization of feature information. Pu L. et al. [22] proposed a novel authentication system based on multi-wavelet decomposition for feature extraction. They transformed PPG signals into a latent space using autoencoders and classified users using different distance metrics. Comparing their approach with CNNs and LSTM networks across three datasets (totaling 120 subjects), they identified the optimal method using GHM wavelets combined with autoencoders, achieving an accuracy of 98%. Despite its excellent performance in identity recognition tasks, this method has limitations. For instance, the multi-wavelet decomposition-based feature extraction, while effective for non-stationary signals, falls short in capturing local signal features. Seok et al. [23] proposed a 1D Siamese neural network model based on PPG signals, which reduces noise through multi-cycle averaging while preserving individual unique features, enabling efficient and secure biometric recognition. However, this method also has limitations. On one hand, the Real-World PPG dataset used in the experiments has low noise levels, and the model’s performance in high-noise environments remains unverified. On the other hand, the dataset’s limited sample size raises questions about the model’s generalization ability on larger datasets. YING, L.C. et al. [24] proposed a Dual-Domain Multi-Scale Fusion Deep Neural Network (DMFDNN). This method primarily consists of a dual-branch deep learning framework for PPG biometric recognition, capable of learning time-varying and multi-scale discriminative features from both time and frequency domains. The model achieved a five-fold cross-validation accuracy of 96% on the Structural Performance Category (SPC) dataset. However, DMFDNN has notable shortcomings. It directly processes 1D PPG signals, and although the dual-branch framework attempts to comprehensively capture information from the time-frequency domain, its direct handling of 1D signals limits its ability to fully exploit the complex and dynamic characteristics of PPG signals.

As demonstrated by previous studies, deep learning has been widely applied to PPG-based identity recognition, offering superior performance compared to traditional machine learning methods. Deep learning models eliminate the need for manually designed feature extractors, thereby reducing reliance on domain expertise. However, most current neural network-based models still face two major challenges. First, existing methods primarily process one-dimensional PPG signals and fail to fully extract and utilize the time-frequency characteristics of the signals, limiting their ability to capture signal complexity and hindering improvements in recognition accuracy. Second, the lack of effective multi-scale feature extraction and fusion mechanisms results in relatively simplistic learned features that cannot adequately represent the critical information within PPG signals.

To enhance the model’s capability in representing the time-frequency features and multi-scale structures of PPG signals, this paper proposes the MSF-SE ResNet50 model, which integrates two-dimensional time-frequency transformation, the SENet attention mechanism, and a Lightweight Multi-Scale Feature Fusion (LMSFF) module. This model optimizes feature representation, focuses on key regions, and incorporates hierarchical feature integration, significantly improving the learning capability for identity-discriminative information. The main contributions are as follows:

1. Two-Dimensional Signal Transformation: We propose a deep learning framework based on 2D transformation. By leveraging the two-dimensional image technique (CWT), we transform one-dimensional PPG signals into two-dimensional time-frequency maps, successfully preserving the time-frequency characteristics of the signals while providing richer spatial information. This approach overcomes the limitations of existing methods in handling complex signals.

2. SENet Attention Mechanism: We introduce the SENet attention mechanism, enabling the model to adaptively focus on the most critical pulse signal features for distinguishing individual identities while suppressing unnecessary noise and interference. This significantly enhances the model’s recognition accuracy.

3. Lightweight Multi-Scale Feature Fusion (LMSFF) Module: We propose a novel Lightweight Multi-Scale Feature Fusion (LMSFF) module and apply it to PPG signal feature extraction. The module achieves multi-scale feature fusion through parallel convolutional operations at different scales, addressing the issue of single-scale feature extraction in existing methods and fully capturing the primary characteristics of the signals.

4. Cross-Stage Feature Fusion Strategy: We adopt a cross-stage feature fusion strategy that leverages the hierarchical feature advantages of the ResNet50 network. This overcomes the limitations of traditional feature fusion methods, enabling efficient learning of both detailed and global patterns in the signals.

## 2. Data

### 2.1. Dataset Construction

The PPG signals used in this study are sourced from the publicly available BIDMC (Beth Israel Deaconess Medical Center) dataset. The BIDMC dataset includes 8 min PPG recordings from 53 individuals, sampled at a frequency of 125 Hz. The age of these 53 participants ranges from 19 to 90 years, including 22 males and 31 females.

**Figure 1:**
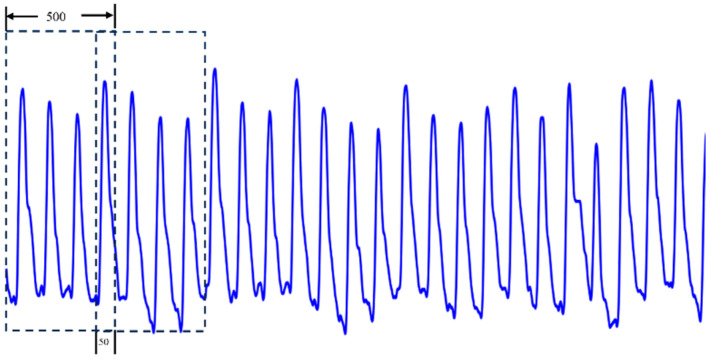
Schematic diagram of overlapping sampling.

To expand the dataset and meet the data requirements for training deep network models, we employed an overlapping sampling method to augment the 53 PPG signals. For each PPG signal, a sliding window [24,25] of length 500 was used to extract segments, with an overlap of 50 sampling points between adjacent windows (a step size of 450 sampling points). The overlapping sampling process is illustrated in Figure 1. Each PPG signal generates 133 segments, resulting in a total of 7049 samples from the 53 individuals.

The task in this study is individual identification, where each individual is treated as a separate class, totaling 53 classes. Each sample is labeled using one-hot encoding to indicate its corresponding individual class. This setup was used for experimental analysis.

### 2.2. K-Fold Cross-Validation

K-fold cross-validation divides the dataset into k subsets, each containing an equal (or nearly equal) number of non-overlapping samples. In each iteration, one subset is used as the test set, while the remaining k-1 subsets are used for training. After k iterations of training and validation, k performance evaluation results are obtained. The final model performance is assessed by averaging these k evaluation results.

### 2.3. Dataset Partitioning

**Figure 2:**
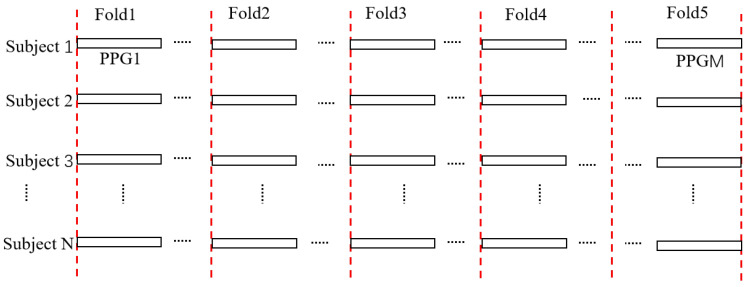
Dataset partition with five folds.

After constructing the dataset, we employed five-fold cross-validation to accurately evaluate model performance. As shown in Figure 2, the dataset is represented as PPG{S1, S2, …, SN}, where SN denotes N individuals, and each individual contains M PPG signals, represented as SN{PPG 1, PPG 2, …, PPGM}. Each individual’s data is then divided into five folds, labeled as {Fold1, Fold 2, …, Fold 5}, with the training set and test set accounting for 80% and 20%, respectively. Since each individual has the same number of PPG signals, the data volume across different classes is balanced, effectively avoiding the issue of class imbalance that could interfere with model training.

### 2.4. Data Preprocessing

Preprocess the PPG signal data based on its characteristics.

Step 1: Denoising. PPG signals are commonly affected by three types of noise interference: (1) electromyographic (EMG) interference, (2) high-frequency random noise, and (3) baseline drift. EMG interference, caused by involuntary muscle tremors, introduces minor errors that are unavoidable but have minimal impact, so they are not extensively studied in this work. A fourth-order Butterworth bandpass filter is employed due to its smooth frequency response characteristics, which minimize amplitude distortion and signal delay during filtering. Considering the characteristics of PPG signals, two critical cutoff frequencies are set for effective filtering. Specifically, the high-pass cutoff frequency is set to 0.5 Hz to effectively eliminate low-frequency noise caused by baseline drift. The low-pass cutoff frequency is set to 8 Hz, which is designed to effectively filter out high-frequency components induced by environmental noise, sensor interference, and other factors. The quality of the filtered PPG signals is significantly improved, laying a solid foundation for subsequent identity recognition tasks.

Step 2: Normalization. Z-score normalization was applied to transform the preprocessed PPG signals into zero mean and unit variance. This method effectively eliminates scale differences between features and facilitates subsequent data analysis and model training. The specific calculation formula is expressed as:

(1) $$z_{i}=\frac{a_{i}-m}{s}$$

In the formula, $a_{i}$ represents the raw PPG signal value, while m and s denote the mean and standard deviation of the PPG signal, respectively.

Step 3: Signal Transformation. Although various deep learning algorithms, such as RNN and LSTM, have been developed specifically for processing one-dimensional time-series data like PPG signals, two-dimensional images offer unique advantages due to their rich spatial information and structural features. By leveraging deep learning methods for feature extraction, detailed information in images, such as edges and corners, can be effectively captured, significantly enhancing learning efficiency. Therefore, transforming one-dimensional PPG signals into two-dimensional images is essential to fully exploit these advantages.

This paper employs the Continuous Wavelet Transform (CWT) to convert one-dimensional PPG signals into two-dimensional images. As a time-frequency analysis technique tailored for non-stationary signals, CWT decomposes signals into localized components across different frequencies, simultaneously providing time and frequency information—a richer analytical perspective compared to traditional time-domain methods. Unlike the Short-Time Fourier Transform (STFT), which uses a fixed window function, CWT utilizes an adjustable window function, enabling flexible balancing of time and frequency resolution. This makes CWT more effective for analyzing non-stationary signals, particularly in capturing transient features and variations.

Through this time-frequency localization property, wavelet transform not only changes the representation of the data but, more importantly, introduces a new dimension to signal analysis. It enables more precise capture of signal variations across different time and frequency scales. This capability allows wavelet transform to reveal detailed information that traditional time-domain methods cannot provide, significantly enhancing the accuracy and depth of signal analysis. For any given signal $x\left(t\right)$, its continuous wavelet transform is defined as:

(2) $$X_{w}\left(u,v\right)=\int_{-\infty }^{\infty }x\left(t\right)\varphi_{u,v}\left(t\right)dt$$

In the formula, u is the translation factor, controlling the position of the wavelet window in the time domain; v is the scaling factor, controlling the size of the wavelet window and its position in the frequency domain; $\varphi_{u,v}\left(t\right)$ is called the wavelet basis function (also known as the mother wavelet), which is expressed as follows:

(3) $$\varphi_{u,v}\left(t\right)=\frac{1}{\sqrt{\left|u\right|}}\varphi \left(\frac{t-v}{u}\right)\;\;\;\;\;\;\;u>0$$

The specific processing steps of the Continuous Wavelet Transform (CWT) are as follows:

(1) Input Data Preparation: The input PPG signal segments are preprocessed through denoising and normalization and then used as the input for CWT.

(2) Wavelet Basis Selection: In this study, the Morlet wavelet is selected as the wavelet basis. The choice of wavelet function is crucial for CWT. The Morlet wavelet excels in analyzing the spectrum of non-stationary signals, achieving an ideal balance between time and frequency resolution. This balance is highly beneficial for feature extraction in deep learning models, thereby improving the efficiency and reliability of model training. Therefore, the Morlet wavelet is chosen for processing PPG signals in this paper.

(3) Scalogram Calculation: The scalogram of the PPG signal is computed using CWT. Each point in the scalogram represents the energy distribution of the signal at a specific time and frequency.

(4) Absolute Value Extraction: To focus on the amplitude of frequency components rather than phase information, the algorithm takes the absolute value of each complex number in the scalogram. This results in a scalogram that clearly displays the intensity variations in the PPG signal across different frequencies.

(5) Scalogram Adjustment: The scalogram is adjusted to match the required image dimensions, ensuring that it can be adapted to the input requirements of subsequent analysis or deep learning models.

The complete process of converting a one-dimensional PPG signal into a two-dimensional time-frequency scalogram using the Continuous Wavelet Transform (CWT) is summarized in Algorithm 1. As discussed in Section 2.4, the selection of CWT and the Morlet wavelet is motivated by their strong capability to analyze non-stationary signals such as PPG.
**Algorithm 1:** One-Dimensional PPG Signal to Two-Dimensional Scalogram via CWT**Input:** 
Preprocessed and normalized PPG signal x(t)
**Output:**$\;\mathrm{Two}-\mathrm{dimensional}\;\mathrm{time}-\mathrm{frequency}\;\mathrm{map}\;(\mathrm{scalogram})\;X_{w}(u,v)$Step 1: Choose mother wavelet ψ(t) = MorletStep 2: For each scale u and time shift v:$\mathrm{Compute}\;X_{w}\left(u,v\right)=\int_{-\infty }^{\infty }x\left(t\right)\varphi_{u,v}\left(t\right)dt$$\mathrm{Step}\;3:\;\mathrm{Take}\;\mathrm{absolute}\;\mathrm{value}:\;S(u,v)=|X_{w}(u,v)|$Step 4: Resize S(u,v) to fixed dimension (224 × 224)

## 3. Methodology

### 3.1. Overall Structure and Process of the Model

For PPG-based identity recognition, considering the characteristics of the dataset, we selected the high-performing ResNet network as the foundation for improvement, specifically ResNet50 [26]. This network, proposed by He et al., incorporates residual connections (Residual Connections), effectively addressing the issues of gradient vanishing and performance degradation in deep networks, enabling efficient training in deep architectures.

The network consists of four major convolutional blocks, each containing multiple small residual modules (bottleneck), specifically 3, 4, 6, and 3 modules, respectively. Each residual module is composed of three convolutional layers: the first layer uses a 1 × 1 convolution for dimensionality reduction, the second layer employs a 3 × 3 convolution for feature extraction, and the third layer applies a 1 × 1 convolution to restore dimensionality. This design, through the introduction of skip connections, facilitates the efficient flow of information, mitigates common training difficulties in deep networks, and ensures effective training.

The third column of Table 1 lists the number of output channels and kernel sizes for each convolutional layer. These values are derived from the standard design of ResNet50 and increase progressively with network depth. Specifically, as the network deepens, the number of output channels gradually increases, allowing the network to capture more complex and abstract feature information. These design choices ensure the network’s superior performance in image classification tasks. When the input image size is 224 × 224, the output sizes of the convolutional blocks are 56 × 56, 28 × 28, 14 × 14, and 7 × 7, respectively. The detailed structure of the ResNet50 network is presented in Table 1.

To extract more comprehensive feature map information, this paper introduces SENet and the lightweight Multi-Scale Feature Fusion (LMSFF) module. The overall process of the model is as follows:

(1) SENet Feature Recalibration

The outputs of the three convolutional blocks in the ResNet50 structure, with sizes of 56 × 56, 28 × 28, and 14 × 14, are fed into the SENet module. SENet performs feature recalibration through squeeze and excitation operations, resulting in Feature Map 1, Feature Map 2, and Feature Map 3.

(2) LMSFF Feature Fusion and Processing

First, Feature Map 1 is input into the LMSFF module. The output feature map undergoes a 1 × 1 convolution and downsampling to match the channel count and dimensions of Feature Map 2. The aligned feature maps are then fused, and the fused result is passed through a ReLU activation function to introduce nonlinearity. The same process is applied to the fusion with Feature Map 3. Finally, the fused result with Feature Map 3 is passed through nonlinearity and input into the LMSFF module. After processing, a 1 × 1 convolution and downsampling are applied to obtain Feature Map A with a size of 7 × 7.

(3) Weighted Fusion

The output of the feature map branch (Feature Map A) is weighted and fused with the output feature map B from the fourth convolutional block of the ResNet50 backbone, resulting in the comprehensive feature map X. The weighted fusion is calculated as:

(4) $$X=\alpha_{1}A+\alpha_{2}B$$

where $\alpha_{1}$ and $\alpha_{2}$ are learnable weighting coefficients.

During model training, $\alpha_{1}$ and $\alpha_{2}$ are initialized with random values and updated iteratively through backpropagation and the optimizer. By optimizing the loss function, the model adaptively adjusts these weighting coefficients, allowing it to automatically determine the relative weights of Feature Map A and Feature Map B at different training stages. This ensures an optimal balance in the contributions of different branches to the final classification result.

(4) Classification Output

The fused result is first processed using global average pooling to convert it into a fixed-size feature vector. A fully connected classifier is then applied to classify the feature vector, ultimately outputting the final identity recognition result.

**Figure 3:**
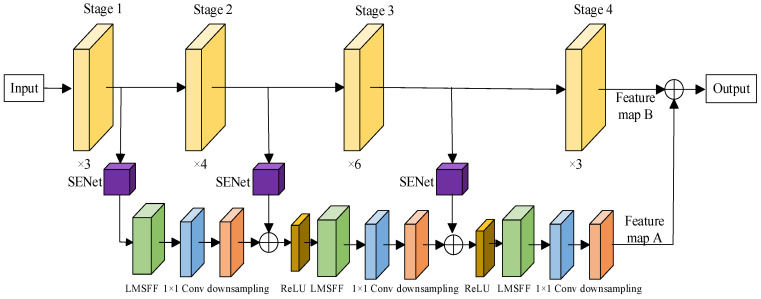
The architecture of the MSF-SE ResNet50 model.

In the proposed model, SENet is introduced first, which enables the model to focus on the most critical pulse signal features for distinguishing between different individuals through adaptive adjustment of feature map weights. Next, the lightweight Multi-Scale Feature Fusion (LMSFF) module is employed, which extracts richer feature map information through parallel processing of convolutions at different scales. Finally, the model adopts a cross-stage feature fusion strategy, effectively integrating the advantages of features from different levels of the ResNet50 network. It leverages high-resolution local features from the low-level network and low-resolution global features from the high-level network, thereby enhancing the model’s ability to capture both detailed signal features and global patterns. The overall architecture of the improved model is illustrated in Figure 3.

**Figure 4:**
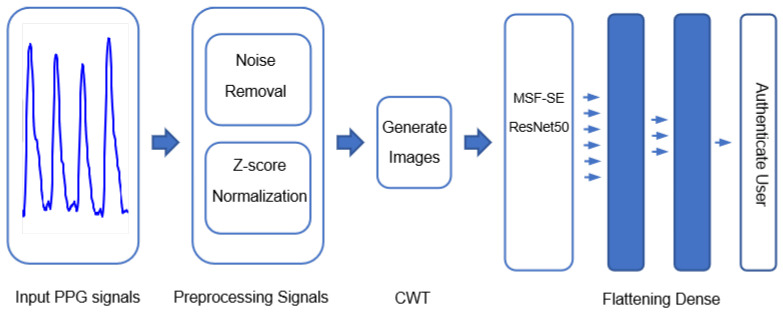
Flowchart of the operation.

The operational flow of the proposed model is shown in Figure 4. First, the raw PPG signal is preprocessed, including denoising and normalization, to improve the signal quality and make it suitable for further analysis. Next, the preprocessed one-dimensional PPG signal is transformed into a two-dimensional time-frequency map using Continuous Wavelet Transform (CWT). Then, the transformed 2D map is input into the MSF-SE ResNet50 model to initiate the training process. Finally, the final classification result is output through a fully connected network layer.

### 3.2. ResNet Network Model

The ResNet network is a milestone in convolutional neural networks. When the number of layers in a neural network is relatively small, increasing the network depth improves the model’s performance. However, as the network depth continues to grow beyond a certain point, issues such as vanishing gradients or exploding gradients may arise. The introduction of ResNet effectively addresses these problems, enabling the model’s performance to continue improving with increased network depth. The most critical component in ResNet is the residual block, whose formula is expressed as:

(5) $$f=g\left(h\right)+h$$

In the formula, h represents the input of the residual block, f represents the output of the residual block, and $g\left(h\right)$ denotes the residual component.

The residual block employs a shortcut connection, which establishes a direct path from the input to the output of the block. During backpropagation, this direct connection allows the network parameters to be updated more effectively, thus avoiding problems such as vanishing gradients or exploding gradients [27]. This design ensures that the training performance of the network remains reliable, even as the network depth increases.

**Figure 5:**
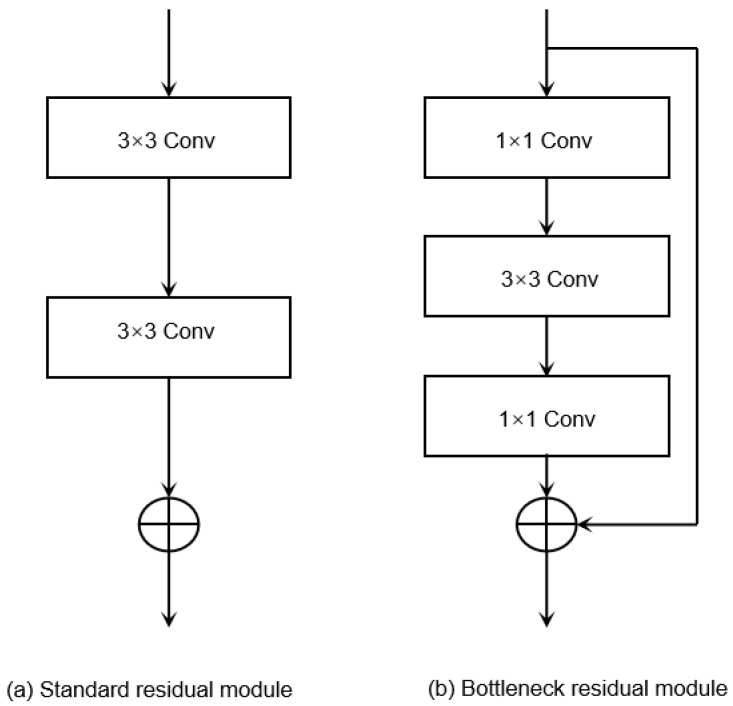
Residual module.

The basic residual module in ResNet mainly has two types. Figure 5a shows the standard residual module, which is typically used in shallower networks like ResNet-18 and ResNet-34. This module consists of two 3 × 3 convolutional layers. Figure 5b depicts the bottleneck residual module, which is applied in deeper networks such as ResNet-50, ResNet-101, and ResNet-152. It includes three convolutional layers: a 1 × 1 convolutional layer for dimensionality reduction, a 3 × 3 convolutional layer for feature extraction, and another 1 × 1 convolutional layer for dimensionality expansion.

### 3.3. SENet Attention

**Figure 6:**
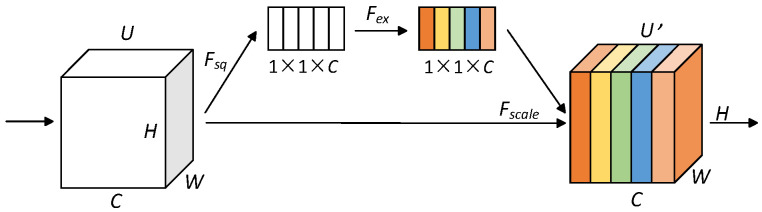
Structure of SENet.

The attention mechanism is a feature-enhancement technique inspired by human attention. Its uniqueness lies in dynamically focusing and allocating attention when processing input data, allowing the neural network to concentrate on more important information [24], thereby assigning weights to features of varying significance. SENet (Squeeze-and-Excitation Network) is a lightweight channel attention mechanism (CAM). It can be seamlessly integrated into neural network models at the cost of minimal additional computation, improving the performance of PPG signal classification. The structure of SENet is shown in Figure 6.

Note: *H* and *W* are the height and width of the feature map, respectively, and *C* is the number of channels; *U* is the set of input feature maps, *U′* is the set of adjusted output feature maps, *F_sq_* is the squeezing operation, *F_ex_* is the excitation operation, and *F_scale_* is the recalibration operation of the feature map.

Before identity recognition tasks, although noise has been removed from the PPG signals, subtle interference may still remain, potentially affecting feature extraction and classification. Embedding a SENet-based channel attention mechanism into the model structure allows the network to automatically focus on important features while ignoring and suppressing irrelevant ones. SENet consists of two key components: squeeze and excitation.

In the squeeze operation, the SENet model performs average pooling on the feature map of each channel, converting it into a scalar value. This scalar represents the overall importance of the features within that channel. By doing so, the SENet model effectively captures the global information of each channel. The squeeze operation is described as:

(6) $$z=\frac{1}{H\times W}\sum_{r=1}^{H}\sum_{c=1}^{W}u_{c}\left(r,c\right)$$

The formula defines z as the result of the squeeze operation, where H and W represent the height and width of the feature map, respectively, and $u_{c}$ is the input matrix. r and c denote rows and columns.

The excitation operation acts as a gating mechanism to adaptively weight the features of each channel. In this process, the squeezed features are passed through a fully connected layer, which includes a ReLU activation function followed by a sigmoid activation function. This design enables the model to automatically learn the importance of features for each channel. The excitation operation is described as:

(7) $$s=\sigma \left(W_{2}\delta \left(W_{1}z\right)\right)$$

In the formula, s represents the excitation result; δ denotes the ReLU activation function; σ represents the sigmoid activation function; $W_{1}\left(\cdot \right)$ and $W_{2}\left(\cdot \right)$ represent two fully connected layer operations, respectively.

### 3.4. Dilated Convolution

Dilated convolution was first introduced by Yu and Koltun (2016), and is also known as atrous convolution. This convolution operation aims to generate higher-resolution feature maps and capture more contextual information over a larger range. Compared to regular convolution, dilated convolution includes an additional hyperparameter known as the dilation rate. The formula for dilated convolution is as follows:

(8) $$n=k+\left(k-1\right)\times \left(d-1\right)$$

(9) $$o=\frac{i-2p-n}{s}+1$$

In the formula, k is the size of the convolutional kernel in the original convolution; d is the dilation rate; n is the size of the effective convolutional kernel in dilated convolution; p is the padding applied during the convolution operation; s is the stride; i is the input size; and o is the output size.

**Figure 7:**
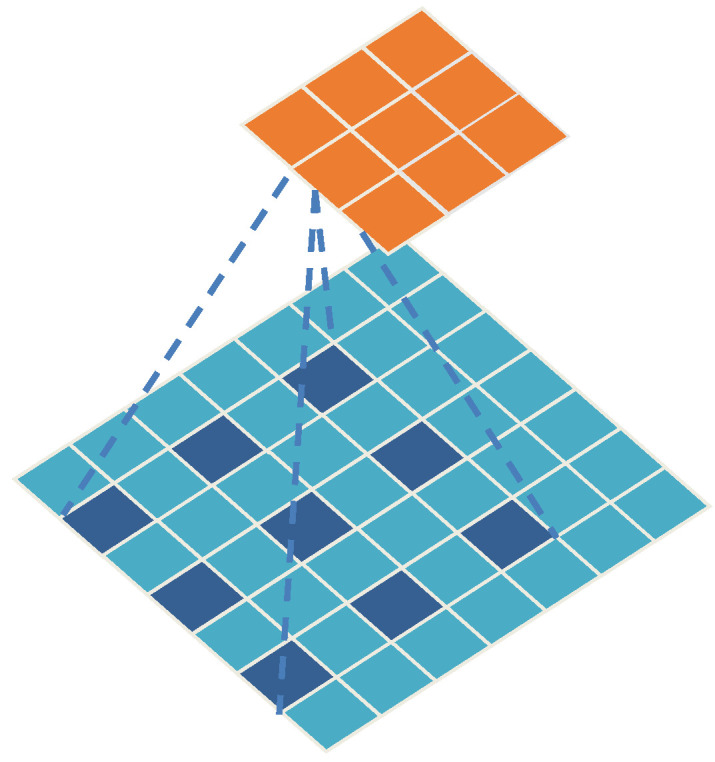
Schematic diagram of dilated convolution.

The size of the dilation rate affects the receptive field of the convolution. Dilated convolution with a larger dilation rate can cover more content information, which is more helpful for understanding larger texture features in an image. On the other hand, dilated convolution with a smaller dilation rate focuses more on the local content of the image, helping to preserve finer details. Figure 7 shows a 3 × 3 dilated convolution kernel with a dilation rate of 2.

### 3.5. Depthwise Separable Convolution Module

In existing research on improving the ResNet50 network model, the model’s accuracy is often enhanced by modifying or adding module structures. However, this typically increases the number of model parameters, which in turn raises the computational cost. To address this, this paper introduces depthwise separable convolution to replace some of the standard convolutions in the original model. This method reduces the number of parameters and computational cost while maintaining model performance, thus improving efficiency.

Compared to regular convolution, depthwise separable convolution significantly reduces the number of parameters and computational cost, enabling lightweight network models while achieving good results in feature extraction. Depthwise separable convolution works by decomposing the standard convolution into two independent steps: depthwise convolution and pointwise convolution [28].

**Figure 8:**
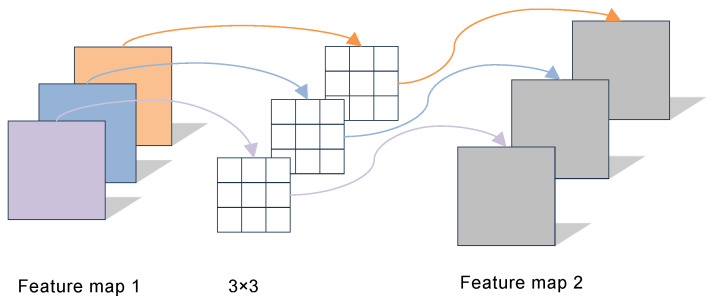
The working principle of depthwise convolution.

Depthwise convolution performs a separate convolution on each channel of the feature map 1 with M channels, producing a single-channel feature map 2 with rich feature information. The working principle of depthwise convolution is illustrated in Figure 8.

**Figure 9:**
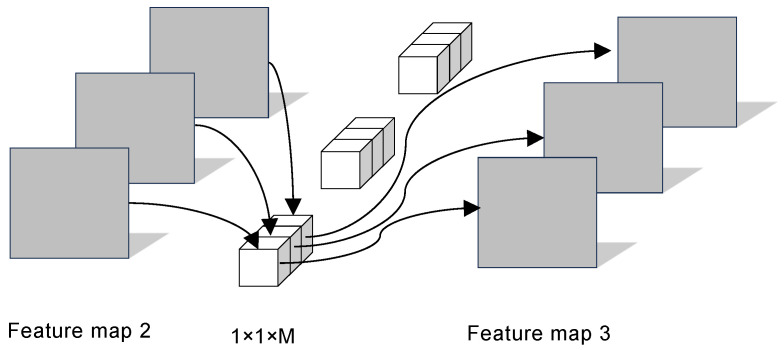
The working principle of pointwise convolution.

Pointwise convolution uses a convolutional kernel with a size of 1 × 1 × M to process the output feature map 2 from the depthwise convolution. Pointwise convolution is used to linearly combine feature information from the same spatial locations across different channels, thus obtaining the final feature map 3, which integrates information from different channels. The working principle of pointwise convolution is illustrated in Figure 9.

### 3.6. Lightweight Multi-Scale Feature Fusion Module (LMSFF)

**Figure 10:**
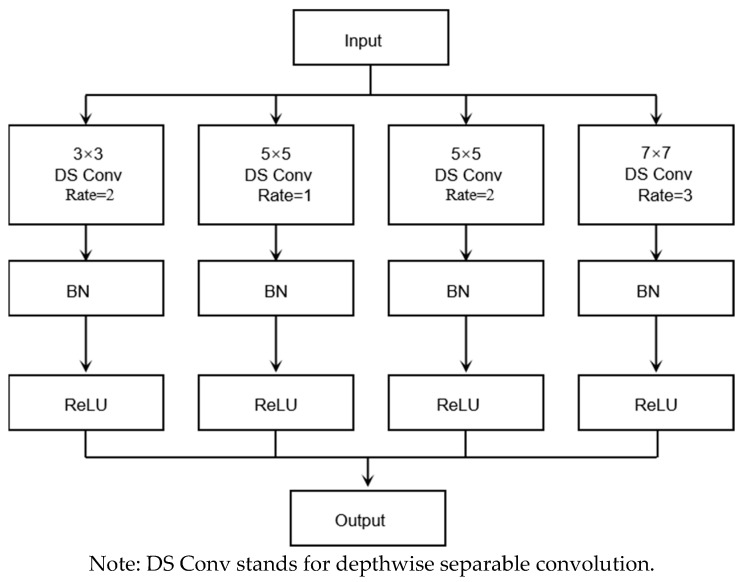
Lightweight Multi-Scale Feature Fusion module.

PPG signals exhibit significant nonlinearity and non-stationarity, primarily due to the physiological characteristics they reflect, such as the dynamic changes in heartbeats and blood flow. Additionally, during actual measurements, PPG signals are susceptible to external factors. For example, variations in light sources (such as ambient light interference, fluctuations in light intensity, and changes in luminous efficiency caused by temperature variations) can lead to instability in signal amplitude. Although commercial systems typically mitigate these effects through techniques like adjusting light source intensity and automatic gain control, some level of interference remains possible. Furthermore, skin properties (such as skin tone and thickness) and variations in the pressure between the sensor and the skin can also impact signal quality. These factors collectively contribute to the rich and complex feature information contained in PPG signals. In ResNet50, due to the fixed size of the convolutional kernels in the residual blocks, feature information can only be extracted at specific scales, which limits its ability to process PPG signals with prominent multi-scale features. Therefore, this paper introduces dilated convolution into the ResNet50 model to enhance feature extraction. By increasing the kernel size, the receptive field is expanded, and different dilation rates are used to extract multi-scale feature information, thus achieving more precise representation of PPG signal features. The receptive field calculation method relevant to this design has already been introduced in Section 3.4. Additionally, to reduce the number of parameters and computational cost, depthwise separable convolution is introduced for lightweight implementation. Building upon this, we propose a Lightweight Multi-Scale Feature Fusion (LMSFF) module, which is designed to effectively capture and integrate the multi-scale characteristics of PPG signals via a parallel architecture, thereby improving the integrity and discriminative capability of the extracted feature representations as shown in Figure 10.

The LMSFF module incorporates the following four distinct convolutional operations, with their specific parameter selections based on the characteristics of PPG signals and a comprehensive consideration of model performance:

First, a 3 × 3 convolution with a dilation rate of 2 is employed to expand the receptive field while capturing small-scale features. This 3 × 3 convolution with a dilation rate of 2 retains local features while incorporating information from a larger surrounding area, enhancing the ability to capture small-scale details. Second, two 5 × 5 convolutions with different dilation rates (1 and 2) are combined to capture medium-scale features. The 5 × 5 convolution with a dilation rate of 1 focuses on medium-scale features while preserving more local details, whereas the 5 × 5 convolution with a dilation rate of 2 further expands the receptive field to extract medium-scale features from a broader range. This combination enables the capture of richer feature information at the medium scale. Compared to 3 × 3 kernels, 5 × 5 kernels cover a larger area. With a dilation rate of 1, the 5 × 5 convolution retains more local fine-grained features, while a dilation rate of 2 extends the range to capture more comprehensive medium-scale features. Together, these operations comprehensively cover the feature variations in PPG signals at this scale. Finally, a 7 × 7 convolution with a dilation rate of 3 is employed to significantly enlarge the receptive field, primarily for capturing large-scale features. This effectively captures the overall trends and periodic changes in PPG signals, which are crucial for understanding the macro-level characteristics and long-term patterns of the signal.

To enhance the network’s generalization ability and accelerate convergence, batch normalization (BN) and ReLU activation layers are applied after each of the proposed four different convolution layers. By employing the above four convolution operations, feature maps with four different receptive fields are obtained, which are subsequently fused at the pixel level to form a multi-scale feature fusion module that is added to the residual neural network. Additionally, to improve computational efficiency while preserving high feature extraction capability, all convolution operations are replaced with depthwise separable convolutions.

To provide a clearer explanation of the implementation process of the LMSFF module, Algorithm 2 summarizes the detailed steps as follows:
**Algorithm 2:** Lightweight Multi-Scale Feature Fusion (LMSFF) Module**Input:** 
$\mathrm{Feature}\;\mathrm{map}\;\mathrm{tensor}\;F\in R^{C\times H\times W}$
**Output:** $\mathrm{Multi}-\mathrm{scale}\;\mathrm{fused}\;\mathrm{feature}\;\mathrm{map}\;F_{fused}\in R^{C^{’}\times H\times W}$Step 1: Multi-Scale Convolutional Feature Extraction:$F_{1}=DSConv_{3\times 3,r=2}\left(F\right)$ $F_{2}=DSConv_{5\times 5,r=1}\left(F\right)$ $F_{3}=DSConv_{5\times 5,r=2}\left(F\right)$ $F_{4}=DSConv_{7\times 7,r=3}\left(F\right)$Step 2: BN + ReLU:Fi←ReLU(BN(Fi)), For i∈1,2,3,4Step 3: Feature Fusion:
$F_{concat}\leftarrow Concat(F_{1},F_{2},F_{3},F_{4})$

## 4. Experimental Results and Discussion

### 4.1. Experimental Setup and Evaluation Metrics

The experimental platform configuration includes 32 GB of RAM, an Intel Core i5-13500H processor with a clock speed of 2.60 GHz, and acceleration using the NVIDIA Tesla P100 GPU provided by Kaggle, with 16 GB of VRAM. The experimental environment runs on the Windows 10 operating system, and the development language used is Python 3.8. The deep learning model is built and trained using the PyTorch 1.10.1 framework.

To comprehensively evaluate the model’s performance, four metrics are chosen: accuracy (Acc), precision (Pre), recall, and F1 score. Their calculation formulas are as follows:

(10) $$Acc=\frac{TP+TN}{TP+TN+FP+FN}\times 100\%$$

(11) $$Pre=\frac{TP}{TP+FP}\times 100\%$$

(12) $$Recall=\frac{TP}{TP+FN}$$

(13) $$F1=\frac{2\times Pre\times Recall}{Pre+Recall}$$

In the formulas, TP represents the number of times the model correctly identifies the true identity of an individual, TN represents the number of times the model correctly identifies that an individual does not belong to a specific identity, FP represents the number of times the model incorrectly classifies an individual as a wrong identity, and FN represents the number of times the model fails to correctly identify the true identity of an individual.

### 4.2. Experimental Parameters

#### 4.2.1. Parameter Sensitivity Analysis

**Figure 11:**
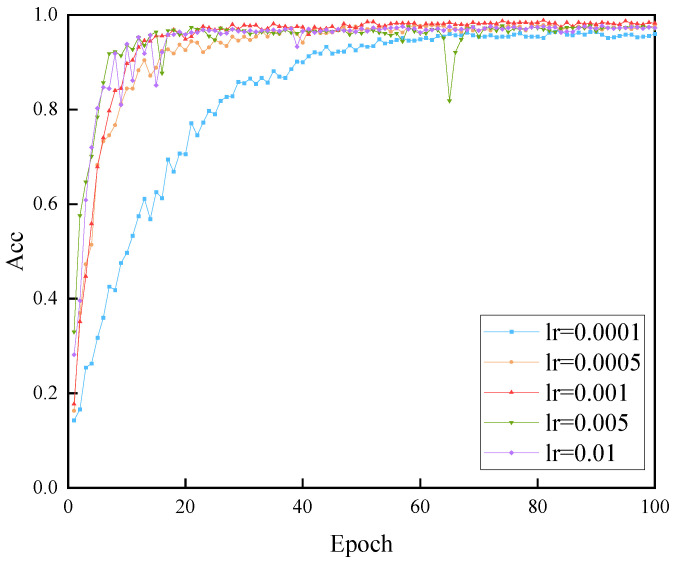
Impact of different learning rates on model performance.

This study investigates the impact of different initial learning rates and batch sizes on the performance of the MSF-SE ResNet50 model. Figure 11 shows the identity recognition accuracy corresponding to different learning rates over 100 network epochs. From the figure, it can be observed that when the learning rate is too high, the accuracy fluctuates significantly. Conversely, when the learning rate is too low, the model converges more slowly, and the recognition accuracy decreases. The model achieves the best overall performance when the learning rate (lr) is set to 0.01. At this learning rate, the recognition accuracy stabilizes after 40 epochs and reaches the highest level.

**Figure 12:**
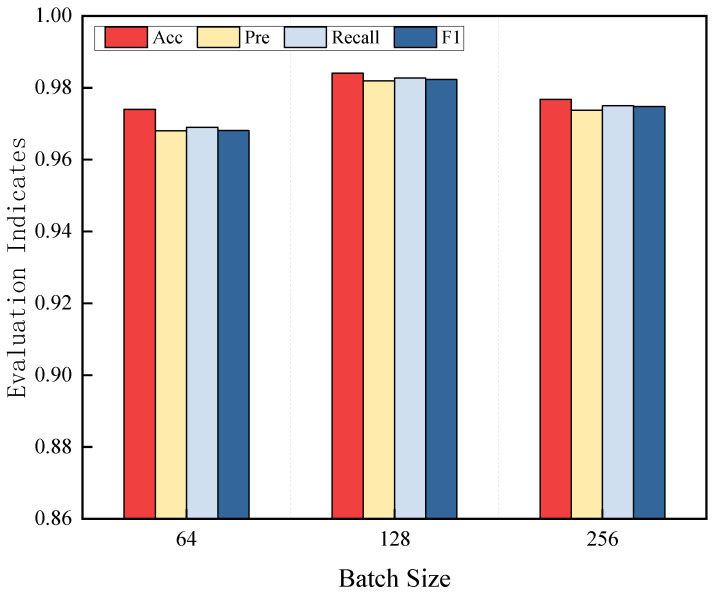
Impact of different batch sizes on model performance.

After fixing the learning rate, the model was trained with batch sizes of 64, 128, and 256, and the performance results are shown in Figure 12. The experimental results indicate that the model achieves the best performance when the batch size is set to 128.

#### 4.2.2. Experimental Parameter Settings

In our experiments, we observed that the model’s performance did not significantly improve after 100 epochs, indicating that the model had sufficiently converged. Therefore, we set the number of epochs to 100 to balance training efficiency and model performance. For optimization, we employed the Adam optimizer, which is widely used and has demonstrated excellent performance in deep learning tasks. Adam combines the advantages of momentum and adaptive learning rates, enabling faster convergence in the early stages of training through a higher learning rate, while automatically reducing the learning rate as it approaches the optimal solution, thereby avoiding oscillations and improving training stability. The initial learning rate was set to 0.001, the batch size to 128, and the loss function to Cross-Entropy Loss.

The Cross-Entropy Loss function is commonly used in deep learning, especially for classification tasks. It is used to measure the difference between the predicted probability distribution and the true label distribution. Specifically, the Cross-Entropy Loss function measures the error by calculating the negative log-likelihood value between the predicted probability distribution and the true distribution of the target label. The closer the predicted probabilities are to the true labels, the smaller the Cross-Entropy Loss value, indicating better model performance. Mathematically, the Cross-Entropy Loss function is defined as:

(14) $$L=-\sum_{i=1}^{n}y_{i}\log(p_{i})$$

In the formula, $y_{i}$ represents the true label of the class, and $p_{i}$ represents the probability predicted by the model for that class.

### 4.3. Experimental Results Analysis

**Figure 13:**
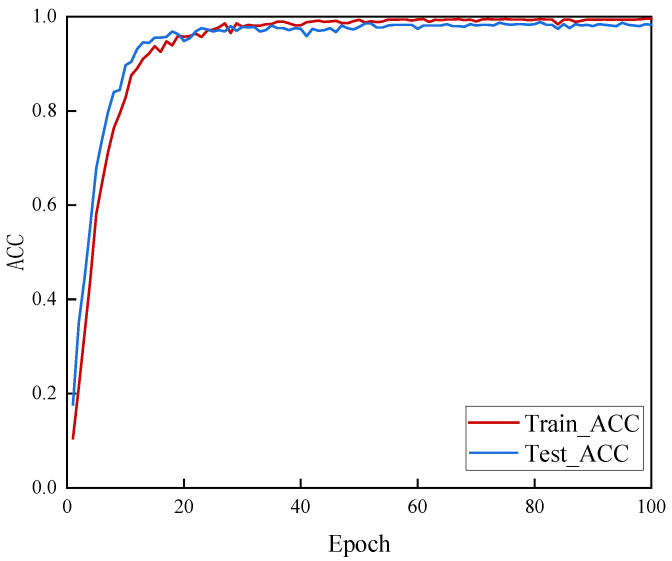
The accuracy curve of the MSF-SE ResNet50.

**Figure 14:**
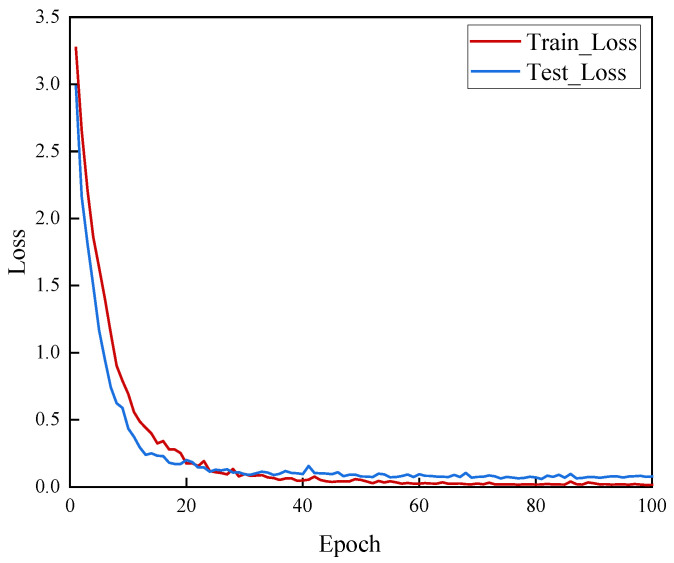
The loss curve of the MSF-SE ResNet50.

Figure 13 and Figure 14, respectively, show the changes in accuracy (Acc) and loss (Loss) on the training set and test set for MSF-SE ResNet50 over 100 training epochs.

From the above results, it can be observed that the model quickly increases accuracy and decreases loss on both the training and test sets. Convergence begins at the 20th epoch, and by the 40th epoch, the model has stabilized. This indicates that the proposed model can effectively learn and capture key features in a short amount of time, reaching convergence quickly. Furthermore, after convergence, the accuracy on the test set remains high and stable, and the loss stays at a low level, further demonstrating the model’s robustness and generalization ability.

Given the high accuracy of the results, it is necessary to analyze the potential risk of overfitting. From a structural perspective, the MSF-SE ResNet50 architecture incorporates batch normalization, which contributes to training stability. Additionally, we employed L2 regularization to prevent excessive model weights, thereby mitigating the risk of overfitting. From a performance perspective, our test set shows stable convergence without the typical overfitting phenomenon where the training loss decreases while the test loss increases. Therefore, there is no evidence of overfitting.

In addition to the high accuracy and rapid convergence demonstrated by the MSF-SE ResNet50 model, we further conducted an in-depth evaluation of its real-time performance to validate its feasibility in practical applications. Real-time performance is crucial for deploying the model in scenarios such as wearable devices or real-time authentication systems. By utilizing the NVIDIA Tesla P100 GPU for computational acceleration, we calculated that the inference time for a single PPG signal (4 s) is approximately 33 ms. This low inference time indicates that the model can efficiently process PPG signals, fully meeting the requirements of real-time applications, and is suitable for deployment in practical scenarios.

### 4.4. Ablation Experiment

To evaluate the performance of the MSF-SE ResNet50 model proposed in this paper, a series of ablation experiments were conducted. The goal of the experiment was to verify whether the various improvements to the model contribute to the enhancement of identity recognition accuracy (Acc), precision (Pre), recall (Recall), and F1 score based on PPG signals.

As mentioned earlier, the improvements to ResNet50 can be summarized in the following two points:

(1) Introduction of attention mechanism modules to recalibrate features.

(2) Introduction of the LMSFF module to achieve multi-scale feature fusion.

Based on the improvement process, ablation experiments were conducted on the expanded PPG signal dataset, and the results of the ablation experiments are shown in Table 2.

From Table 2, it can be observed that the unmodified ResNet50 model has the lowest performance across all metrics, with an accuracy of 94.10%, precision of 94.03%, recall of 93.95%, and F1 score of 93.99%. When only SENet is added, the model’s recognition accuracy increases to 95.22%, which is a 1.12% improvement compared to ResNet50. This result validates that the attention mechanism helps suppress irrelevant features during feature extraction, allowing the model to focus on the most relevant features for classification, thus improving classification performance. When only the multi-scale feature fusion module (LMSFF) is incorporated, the model achieves a recognition accuracy of 97.47%, representing a 3.37% improvement over ResNet50. Additionally, precision, recall, and F1 score all show enhancements, demonstrating the effectiveness of the proposed LMSFF module. Finally, when both SENet and the multi-scale feature fusion module (LMSFF) are introduced, all metrics reach their maximum values, with an accuracy of 98.41%, precision of 98.19%, recall of 98.27%, and F1 score of 98.23%. These results show that the overall performance of the proposed method significantly improves compared to the original ResNet50 model.

### 4.5. Comparison with One-Dimensional Neural Networks

The MSF-SE ResNet50 model proposed in this paper uses two-dimensional image data transformed by Continuous Wavelet Transform (CWT) as input. To validate the effectiveness of the CWT-based two-dimensional image input method, this study selected three neural network models—1D-CNN, LSTM, and Transformer—that are widely used for processing time-series data and capture temporal dependencies in different ways, as the baseline models for comparison. The 1D-CNN [29] can extract local features from PPG signals through convolutional operations. Although it does not explicitly model long-term temporal dependencies, it excels at capturing short-term variations in signals. LSTM [30], as a variant of recurrent neural networks (RNNs) capable of capturing long-term temporal dependencies, effectively models temporal features in PPG signals, making it suitable for handling dynamic changes in the data. This allows us to evaluate the contribution of temporal modeling methods to identity recognition tasks. The Transformer model [31] employs a self-attention mechanism, demonstrating strong performance in processing long time-series data. It captures global dependencies and improves efficiency through parallel computation. By comparing these models, we comprehensively evaluate the performance of different network architectures in PPG signal processing, further validating the advantages of using CWT-transformed 2D image inputs.

The comparison process uses the same dataset and computational environment. However, different model structures require different hyperparameter settings to achieve optimal performance on the same dataset. Therefore, our hyperparameter settings are not identical across models. For each baseline model, we adjusted the hyperparameters to achieve the best performance. Specifically, we referenced the hyperparameter settings from the original papers of each baseline model and experimented with additional settings tailored to our study, selecting the best results for performance comparison. Table 3 presents the performance of these four networks in terms of accuracy (Acc/%), precision (Pre/%), recall (Recall/%), and F1 score (F1/%).

From Table 2, it is evident that the MSF-SE ResNet50 model proposed in this paper surpasses traditional one-dimensional neural network models (1D-CNN, LSTM, Transformer) in all performance metrics. Among them, the 1D-CNN and LSTM models have relatively low performance across all metrics, while the Transformer model shows some improvement but still performs much worse than the MSF-SE ResNet50 model. This result not only verifies the excellent performance of the proposed model but also demonstrates the effectiveness of the CWT-based two-dimensional image input method. By more effectively extracting and utilizing the key features in the data, this method enhances the model performance in identity recognition tasks. Furthermore, it provides further evidence of the great potential and value of transforming data representations to improve model performance in the field of signal processing.

### 4.6. Performance Comparison with Two-Dimensional Neural Networks

To comprehensively evaluate the performance of the MSF-SE ResNet50 model, this paper compares it with six mainstream two-dimensional neural networks. VGG-19 [32] and GoogLeNet [33], as classic deep convolutional neural network models, possess strong feature extraction capabilities and provide a benchmark for the performance of traditional convolutional networks on this task. CNN-RNN [20] and CNN-LSTM [21] combine the strengths of convolutional neural networks (CNNs) and recurrent neural networks (RNNs or LSTM). The CNN component extracts spatial features, while the RNN or LSTM component captures the dynamic changes and temporal dependencies in PPG signals, thereby better adapting to the complex characteristics of PPG signals. ShuffleNet [34], a lightweight network, offers high computational efficiency and is well-suited for resource-constrained environments. DenseNet [35] improves feature reuse and gradient flow through dense connections, enhancing model performance. All these models use two-dimensional image data generated from one-dimensional time-series data via Continuous Wavelet Transform (CWT) as input. Here, we also reference the hyperparameter settings from the original papers of each baseline model and experiment with additional settings tailored to our problem, selecting the best results for performance comparison. Table 4 presents the performance comparison results of these models in terms of accuracy (Acc/%), precision (Pre/%), recall (Recall/%), and F1 score (F1/%).

Based on the experimental results in Table 4, it is clear that the method proposed in this paper outperforms the other six models in terms of accuracy, precision, recall, and F1 score. This advantage is attributed to the model’s strong feature extraction capability, particularly its ability to extract more refined features from the two-dimensional image data generated by Continuous Wavelet Transform (CWT). By incorporating SENet and the LMSFF module, MSF-SE ResNet50 can effectively capture the key information in the CWT-generated 2D images, thus demonstrating higher accuracy and better generalization ability in the identity recognition task.

### 4.7. Comparison with Existing State-of-the-Art Models

To further validate the performance of the MSF-SE ResNet50 model, we compare it with several state-of-the-art methods, all using the BIDMC dataset. The results are shown in Table 5. In [36], a deep autoencoder is used to extract features from PPG signals, followed by a Local Outlier Factor (LOF) algorithm for user authentication, achieving an accuracy of 91.80%. In [37], features extracted using a hybrid convolutional vision transformer (CVT) and convolutional mixer (ConvMixer) are fused, and an attention mechanism is employed for identity recognition, achieving a classification accuracy of 95.00%. In [38], an LW-CNN model consisting of an initial convolutional layer, three residual blocks, a global average pooling layer, a dropout layer, and a fully connected layer is proposed, achieving an accuracy of 96.17% after 80 epochs. In [39], scalable end-to-end 1D convolutional neural networks are employed to extract features from PPG signals, incorporating strategies such as stacked convolutions, non-local blocks, and attention mechanisms. The method achieves an accuracy of 96.46% on the BIDMC dataset. In [40], a secure PPG-based authentication method using homomorphic encryption and a Homomorphic Random Forest classifier is proposed, achieving a classification accuracy of 97.2%. In [25], a PPG-based identity recognition model named PulseID is proposed, which generates multi-scale signals through PPG data augmentation and integrates a feature fusion module along with a curriculum learning training strategy. The model ultimately achieves a classification accuracy of 97.80%.

In comparison, the MSF-SE ResNet50 shows significant improvement on the same dataset, achieving an accuracy of 98.41%, which is 6.61, 3.41, 2.24, 1.95, 1.21, and 0.61 percentage points higher than those of [36], [37], [38], [39], [40], and [25] respectively. Additionally, the MSF-SE ResNet50 model outperforms other network architectures across all other metrics. These results demonstrate that the MSF-SE ResNet50 has stronger feature extraction capabilities and higher classification performance when processing PPG signals, enabling more accurate individual identity recognition.

## 5. Conclusions

In the field of PPG-based identity recognition, the long-standing challenges include the lack of distinctive features and low classification accuracy, with traditional methods facing bottlenecks in feature extraction and performance improvement. To address these issues, this paper proposes a novel network architecture—MSF-SE ResNet50. The network takes two-dimensional PPG signals obtained through Continuous Wavelet Transform (CWT) as input, leveraging the strengths of the SENet module and the Lightweight Multi-Scale Feature Fusion (LMSFF) module, combined with cross-stage feature fusion technology. This enables the model to achieve stronger feature extraction capabilities, enhanced information fusion, and significant recognition accuracy. Ablation experiments have validated the effectiveness of incorporating the SENet and LMSFF modules. Additionally, we selected six high-performing deep learning models (VGG-19, GoogLeNet, CNN-RNN, ShuffleNet, DenseNet, and CNN-LSTM) as baseline models for comparative experiments. Experimental validation based on the BIDMC dataset shows that the proposed MSF-SE ResNet50 model achieved an accuracy (Acc) of 98.41%, a precision (Pre) of 98.19%, a recall of 98.27%, and an F1 score of 98.23%, significantly outperforming all baseline models. In addition, we conducted performance comparisons with several existing state-of-the-art methods on the same dataset. The results demonstrate that MSF-SE ResNet50 consistently achieved the best performance. These findings further validate the proposed model’s advantages in feature extraction and individual identification accuracy, highlighting its strong potential for both research and practical applications in PPG-based biometric recognition. To evaluate the real-time performance of the model, we also measured its inference time during the forward propagation phase. The results show that the inference time for a 4 s PPG signal is approximately 33 milliseconds. This low inference time indicates that the model can process PPG signals in real-time, making it suitable for deployment in practical applications. Essentially, this research bridges the fields of biometric authentication, PPG signal processing, and deep learning. The high recognition accuracy of the proposed model has broad potential for practical applications, particularly in areas such as biometric authentication, smart healthcare, and remote monitoring.

Based on the challenges and limitations of this study, future research directions will provide important momentum for the further development of PPG-based biometric authentication and deep learning technologies. The following are detailed future research directions:

1. To better capture the physiological characteristics of PPG signals and further enhance model performance, we plan to explore the design of a customized wavelet basis function tailored to the unique shape of PPG signals.

2. Investigate effective model compression and lightweight design to reduce model complexity.

3. Introduce more diverse datasets (e.g., MIMIC) to validate the algorithm’s generalization ability across different scenarios and populations, further optimizing the model’s robustness.

4. Future research will focus on the energy-efficient execution of the algorithm on wearable devices in real-time environments. Specifically, we will explore the deployment of the algorithm on microcontrollers or mobile platforms (e.g., Android) using the schemes proposed in references [41,42]. Additionally, we will consider hardware solutions such as Application-Specific Integrated Circuits (ASICs) or System-on-Chip (SoC) to further enhance the algorithm’s execution efficiency.

## Figures and Tables

**Table 1:** The structure of ResNet50.

Stage	Output Size	Specific Layer
Stage 1	56 × 56	$\begin{array}{l} \left[\begin{array}{c} 1\times 1,64\\ 3\times 3,64\\ 1\times 1,256 \end{array}\right]\times 3 \end{array}$
Stage 2	28 × 28	$\begin{array}{l} \left[\begin{array}{c} 1\times 1,128\\ 3\times 3,128\\ 1\times 1,512 \end{array}\right]\times 4 \end{array}$
Stage 3	14 × 14	$\begin{array}{l} \left[\begin{array}{c} 1\times 1,256\\ 3\times 3,256\\ 1\times 1,1024 \end{array}\right]\times 6 \end{array}$
Stage 4	7 × 7	$\begin{array}{l} \left[\begin{array}{c} 1\times 1,512\\ 3\times 3,512\\ 1\times 1,2048 \end{array}\right]\times 3 \end{array}$

**Table 2:** Ablation study results.

Dataset	Model	SE	LMSFF	Acc/%	Pre/%	Recall/%	F1/%
BIDMC	ResNet50	-	-	94.10	94.03	93.95	93.99
	SE-ResNet50	√	-	95.22	95.14	94.96	95.04
	LMSFF-ResNet50	-	√	97.47	97.62	97.47	97.53
	MSF-SE ResNet50	√	√	98.41	98.19	98.27	98.23

Note: In the table, "√" means the model includes the corresponding module, and "-" means the model does not include the corresponding module.

**Table 3:** Performance comparison between the proposed model and one-dimensional neural networks.

Model	Acc/%	Pre/%	Recall/%	F1/%
1D-CNN [29]	91.42	91.36	91.21	91.28
LSTM [30]	91.53	91.46	91.32	91.39
Transformer [31]	92.74	93.49	92.87	92.85
MSF-SE ResNet50 (ours)	98.41	98.19	98.27	98.23

**Table 4:** Performance comparison between the proposed model and two-dimensional neural networks.

Model	Acc/%	Pre/%	Recall/%	F1/%
ShuffleNet [34]	91.90	92.34	91.57	91.93
VGG-19 [32]	93.33	93.50	93.14	93.32
GoogLeNet [33]	93.61	93.29	92.28	92.72
CNN-RNN [20]	94.81	92.49	92.43	92.27
DenseNet [35]	95.15	93.36	92.69	92.88
CNN-LSTM [21]	95.77	96.27	94.21	93.88
MSF-SE ResNet50 (ours)	98.41	98.19	98.27	98.23

**Table 5:** Performance comparison between the proposed model and existing state-of-the-art models.

Dataset	Studies	Acc/%	Pre/%	Recall/%	F1/%
BIDMC	Aly [36]	91.80	95.90	88.30	91.00
	Ibrahim [37]	95.00	97.00	92.00	93.00
	Zhao [38]	96.17	96.24	96.28	96.11
	Wang [39]	96.46	96.21	96.09	96.15
	Zhang [40]	97.20	-	-	-
	Wei [25]	97.80	-	-	-
	MSF-SE ResNet50 (ours)	98.41	98.19	98.27	98.23

## Data Availability

The PPG dataset supporting the findings of this study is available at: https://physionet.org/content/bidmc/1.0.0/ (accessed on 4 June 2025).
